# Supplementary material for: Organic carbon burial in global lakes and reservoirs
Source: Nat Commun. 2017 Nov 22;8:1694. doi: 10.1038/s41467-017-01789-6 (PMC5698497; doi:10.1038/s41467-017-01789-6)
Supplement: Supplementary file 1 — Supplementary Information [file 41467_2017_1789_MOESM1_ESM.pdf]

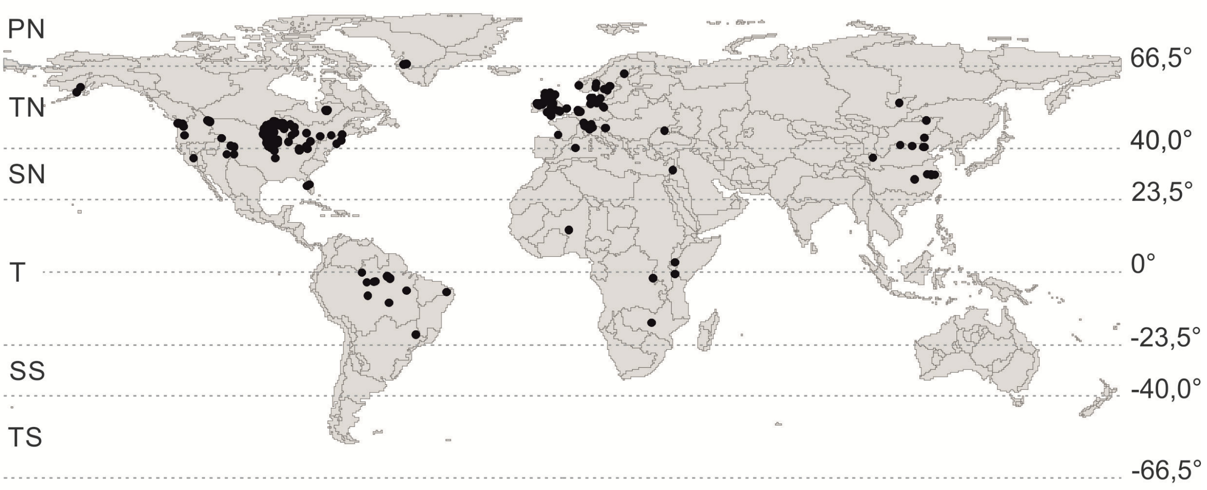

Supplementary Figure 1. Spatial distribution of direct measurements of modern (last ~150 yr) OC burial in lakes and reservoirs. Each circle corresponds to one sampled system; this map shows only the systems for which the geographic coordinates were available in the literature or were found by the authors (368 out of the 403 systems in our literature review). The dashed lines represent reference latitudes that limit the main geographic zones: northern polar (PN), northern temperate (TN), northern subtropical (SN), tropical (T), southern subtropical (SS), southern temperate (TS).

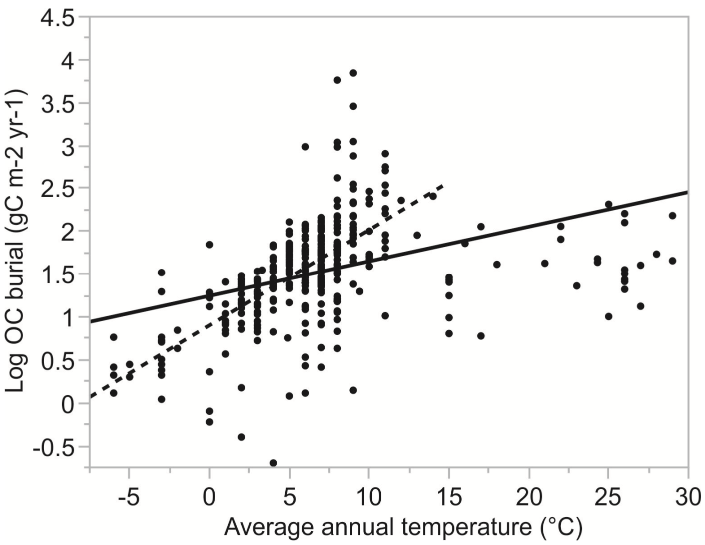

Supplementary Figure 2. Relationship between OC burial rates and average annual air temperatures in each system's watershed. Each circle corresponds to one sampled system; the solid line represents the linear fit including all systems (n=388, R^2adj=0.15, RMSE= 0.54, p<0.0001 , y = 0.04x + 1.24); the dashed line represents the linear fit including only systems with temperatures <15°C (n=358, R^2adj=0.40, RMSE= 0.46, p<0.0001, y = 0.11x + 0.90).

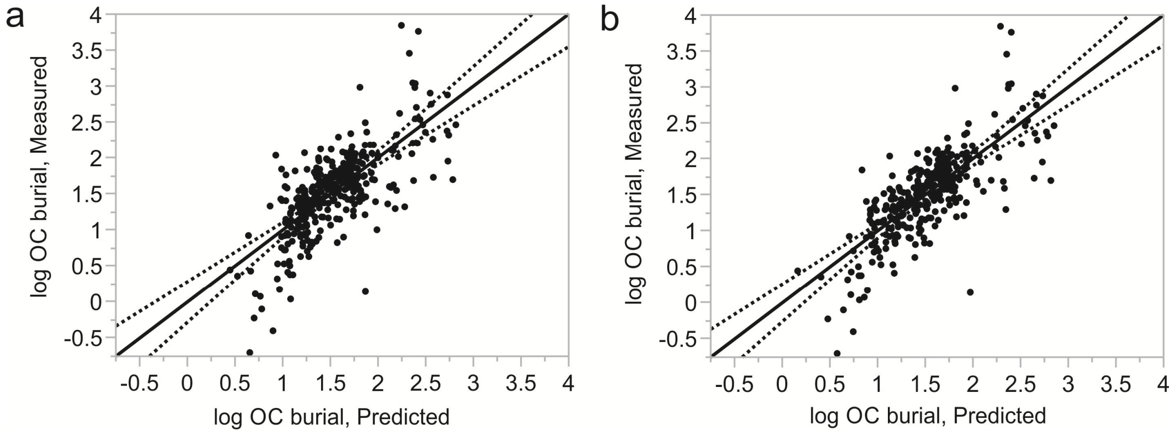

Supplementary Figure 3. Multiple regression models. a, Model 1, including all systems from the literature review (n=362, R^2adj=0.51, RMSE=0.40; p<0.0001); **b,** Model 2, including only systems located in regions of average annual temperature lower than <15°C (n=334, R^2adj=0.57, RMSE=0.38 p<0.0001). Solid lines show best fit of regression; dashed lines indicate 95% confidence interval of best-fit line.

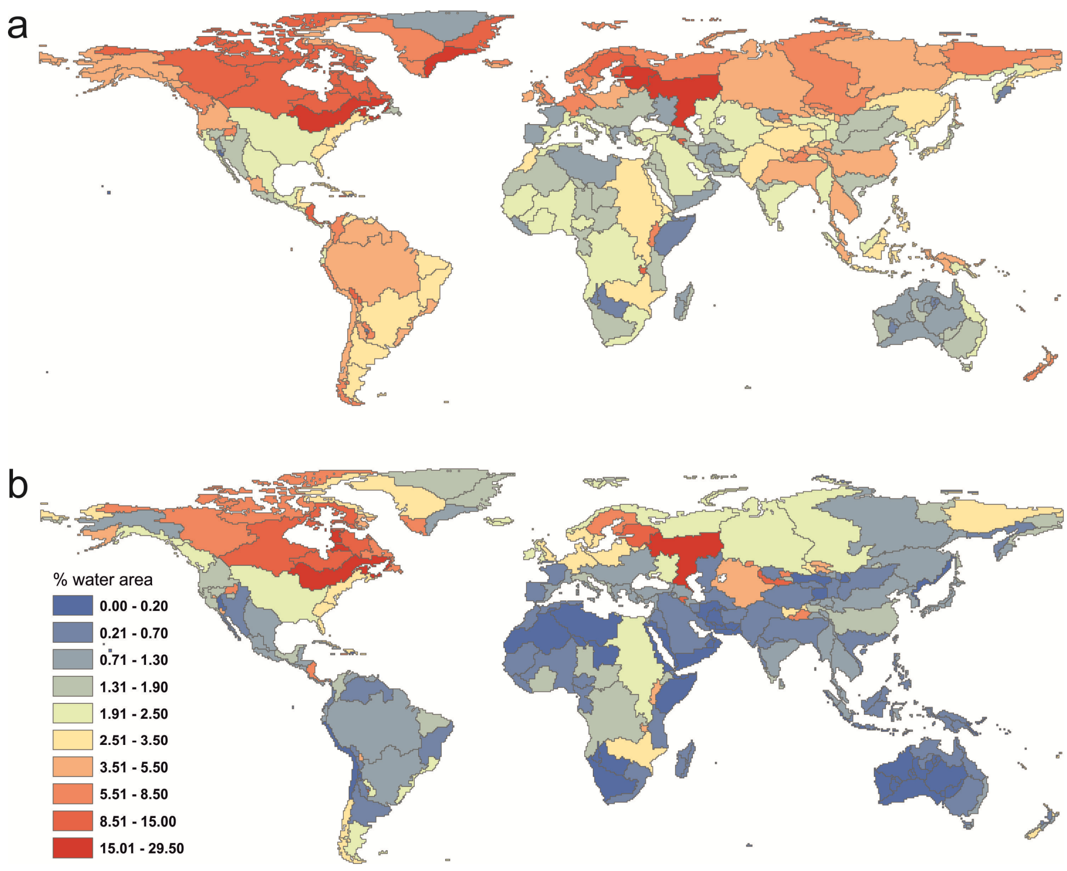

Supplementary Figure 4. Percentage of total water body area in each COSCAT, based on **(a)** the GLOWABO lake inventory[1] and **(b)** the inventory by Raymond *et al.*[2]. This percentage was calculated as the ratio between total lake area and COSCAT area.

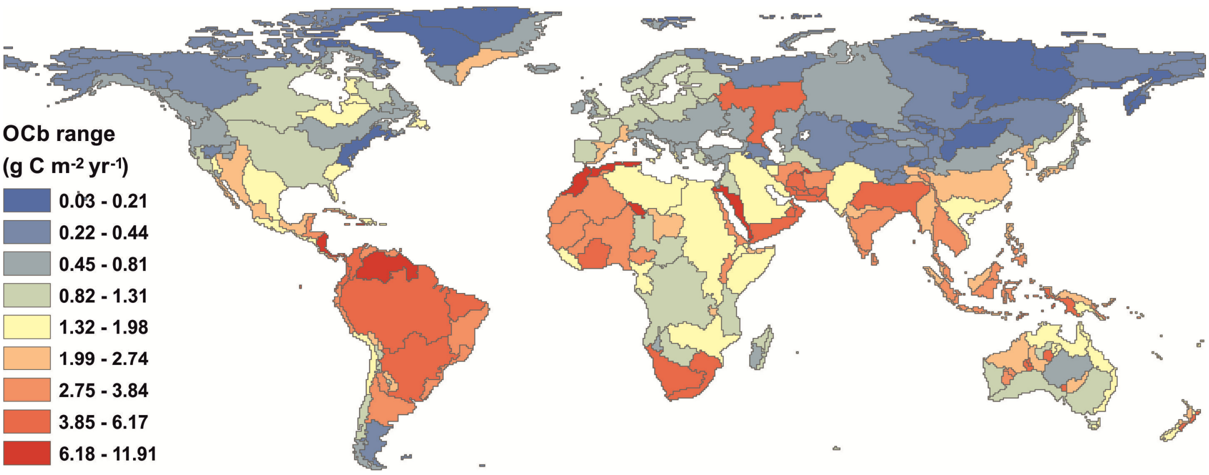

Supplementary Figure 5. Range of OC burial rate per total COSCAT area, from the four scenarios (as in main text, Fig. 2). It was calculated by subtracting the minimum from the maximum estimates of OC burial rate per COSCAT area from the four scenarios.

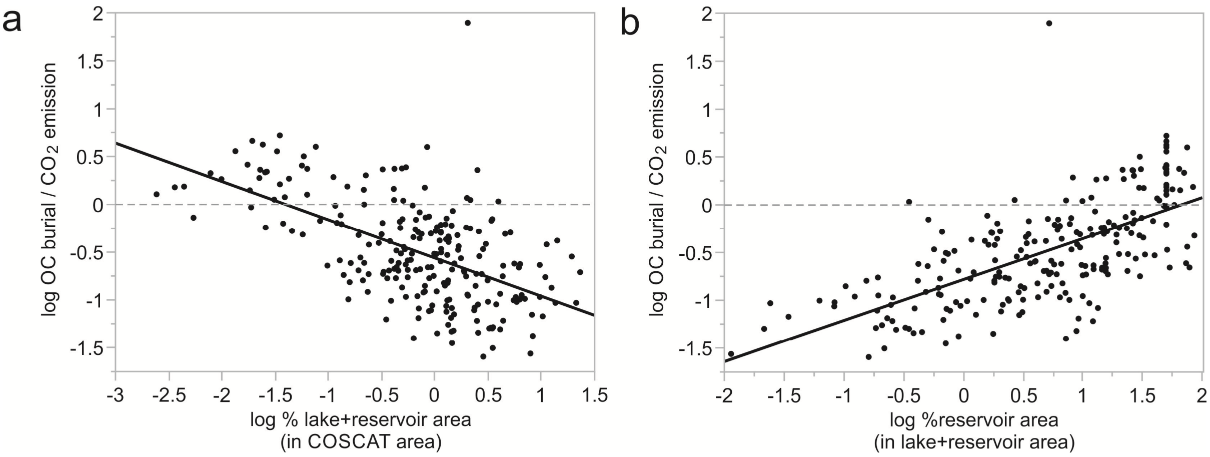

Supplementary Figure 6. Relationship between OC burial / CO_2 emission ratio and (a) percentage lake + reservoir area in total COSCAT area and (b) percentage reservoir area in total lake + reservoir area. Each data point represents one COSCAT; solid lines represent linear fit (**a**, n=230, R^2adj=0. 32, RMSE=0.44, p<0.0001, y = -0.40x - 0.56; **b**, n=230, R^2adj=0.43, RMSE=0.40, p<0.0001, y = 0.43x - 0.78). Points above the horizontal dashed line represent OC burial higher than CO_2 emission. CO_2 emission data is derived from Raymond *et al.*[2]; OC burial rates correspond to scenario AR (see main text).

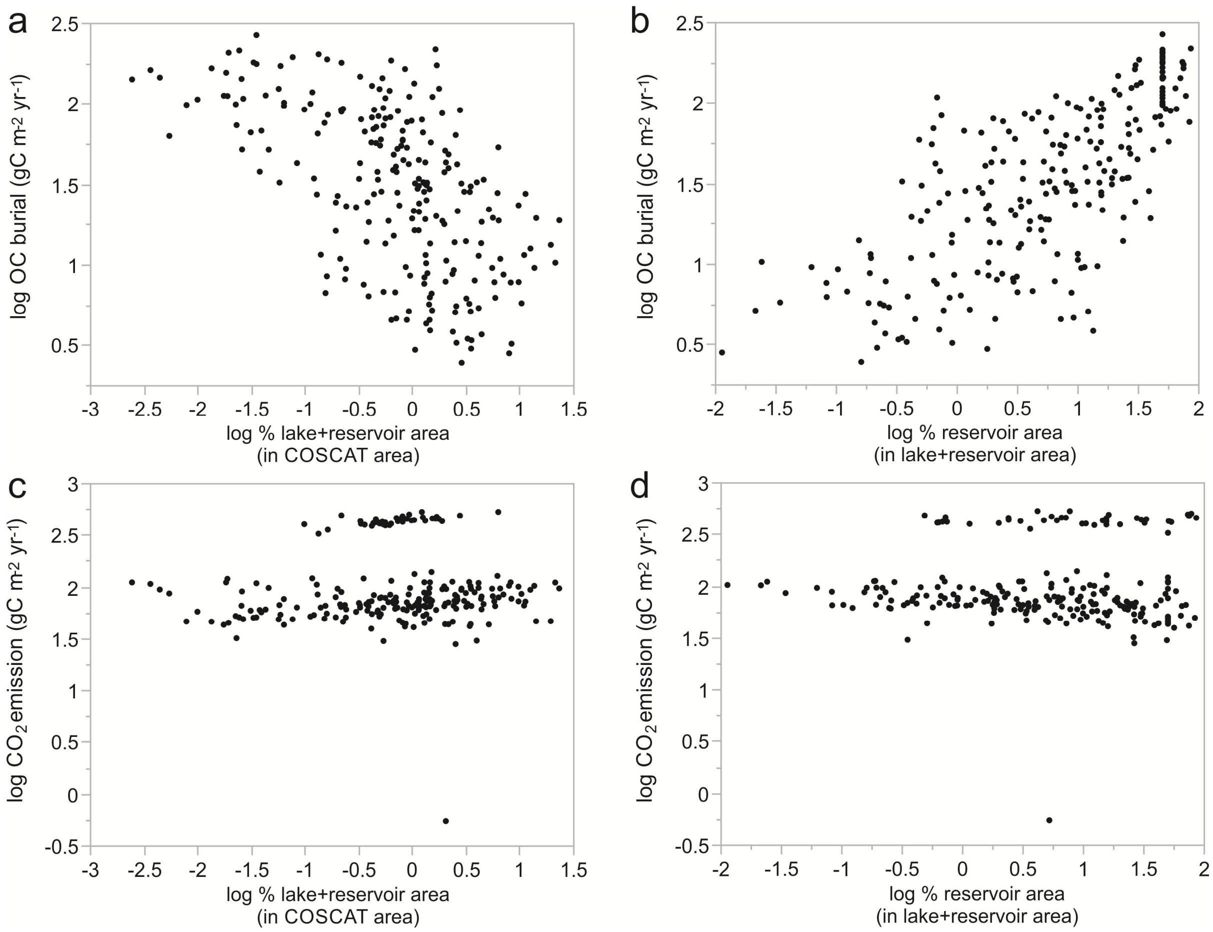

Supplementary Figure 7. Relationship between C fluxes (a and b, OC burial; c and d, CO_2 emission) and percentage inland water area. On the x-axis: a and c, percentage of lake and reservoir area in the total COSCAT area; b and d, percentage of reservoir area in the total lake and reservoir area. Each data point represents one COSCAT; **a,** n=231, R^2adj=0.33, RMSE=0.41, p<0.0001, y = -0.38x + 1.41; **b,** n=231, R^2adj=0.50, RMSE=0.36, p<0.0001, y = 0.43x + 1.19; **c** and **d**, not significant. CO_2 emission data is derived from Raymond *et al.*[2]; OC burial rates correspond to scenario AR (see main text).

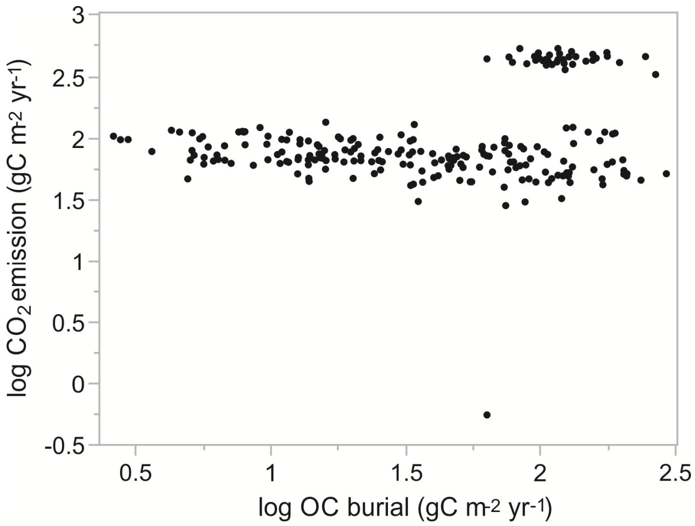

Supplementary Figure 8. Relationship between OC burial and CO_2 emission rates per unit of water body area. Each data point represents a COSCAT; CO_2 emission data is derived from Raymond *et al.*[2]; OC burial rates correspond to scenario AR (see main text).

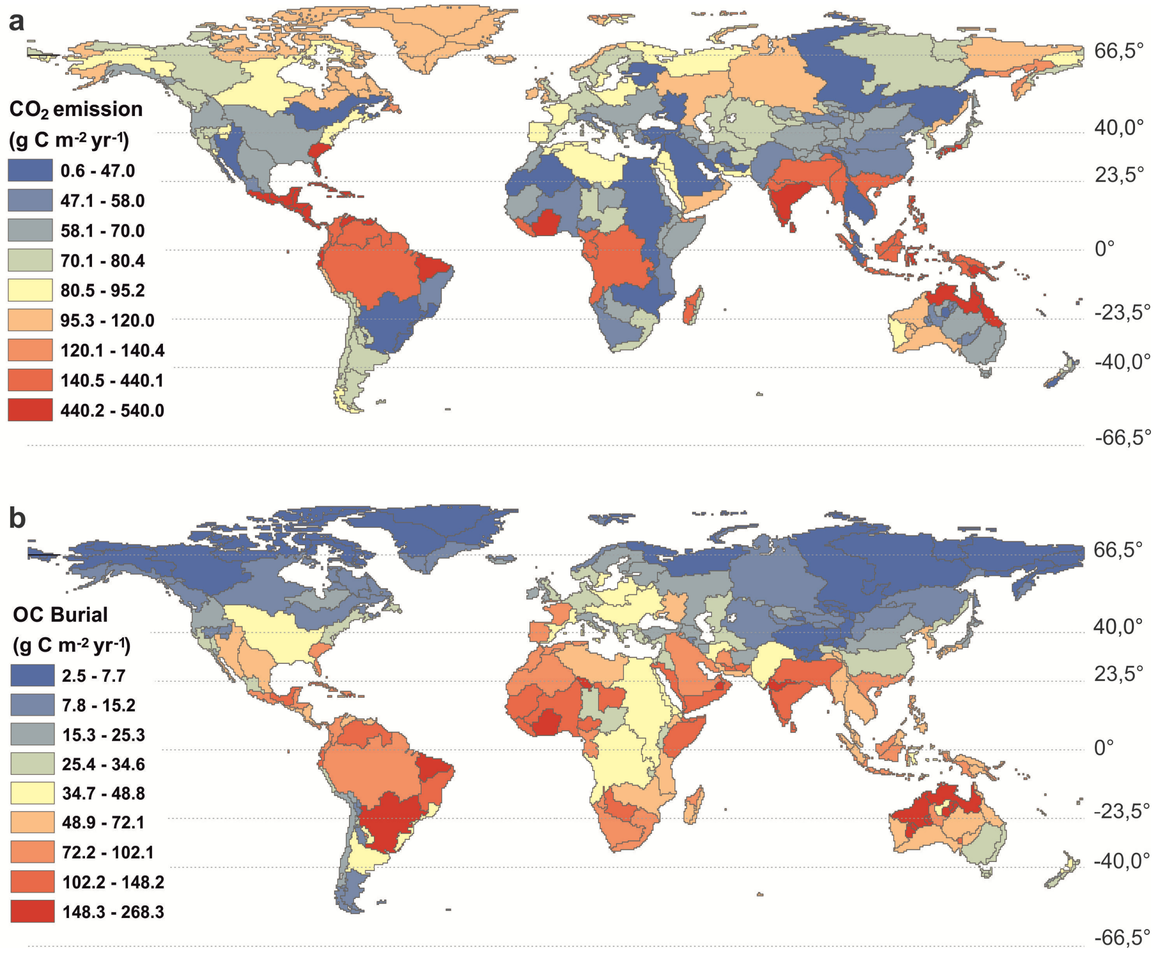

Supplementary Figure 9. CO_2 emission (a) and OC burial (b) rates per unit of water body area. CO_2 emissions are derived from Raymond *et al.*[2]; OC burial rates correspond to scenario AR (see main text).

Supplementary Table 1. Equations to predict Log of OC burial (gC m^{-2} yr^{-1}) from Models 1 and 2. Model 1: Log OC burial = 1.307 + 0.105*Cr - 0.330*Sl - 0.142*Ar + 0.134*Ru + 0.027*Tp ± 0.310 [- if lake, + if reservoir]; n=362, R^2adj=0.51, RMSE=0.40; p<0.0001. Model 2: Log OC burial = 1.040 + 0.065*Cr - 0.307*Sl - 0.124*Ar + 0.146*Ru + 0.069*Tp ± 0.275 [- if lake, + if reservoir]; n=334, R^2adj=0.57, RMSE=0.38 p<0.0001.

Term	Model 1		Model 2	
	Estimate	p-value	Estimate	p-value
Intercept	1.307	<.0001	1.040	<.0001
Cr: Log cropland (%)	0.105	<.0001	0.065	0.0019
Sl: Log average slope (degrees)	-0.330	<.0001	-0.307	<.0001
Ar: Log lake area (km2)	-0.142	<.0001	-0.124	<.0001
Ru: Log runoff (mm yr-1)	0.134	0.0002	0.146	<.0001
Tp: Annual average temperature (°C)	0.027	<.0001	0.069	<.0001
Type [if lake]	-0.310	<.0001	-0.275	<.0001
Type [if reservoir]	0.310	<.0001	0.275	<.0001

Supplementary References

1 Verpoorter, C., Kutser, T., Seekell, D. A. & Tranvik, L. J. A global inventory of lakes based on high-resolution satellite imagery. *Geophys. Res. Lett.* 41, 6396-6402 (2014).

2 Raymond, P. A. *et al.* Global carbon dioxide emissions from inland waters. *Nature* 503, 355-359 (2013).
