# Supplementary material for: Organic carbon burial in global lakes and reservoirs
Source: Nat Commun. 2017 Nov 22;8:1694. doi: 10.1038/s41467-017-01789-6 (PMC5698497; doi:10.1038/s41467-017-01789-6)
Supplement: Supplementary file 3 — Description of Additional Supplementary Files [file 41467_2017_1789_MOESM3_ESM.pdf]

Description of Additional Supplementary Files

File Name: Supplementary Data 1
Description: Modern organic carbon burial rates and watershed characteristics in the lakes and reservoirs from our literature review
